# Investigating on the Methodology Effect When Evaluating Lucid Dream

**DOI:** 10.3389/fpsyg.2016.01306

**Published:** 2016-08-30

**Authors:** Nicolas Ribeiro, Yannick Gounden, Véronique Quaglino

**Affiliations:** CRP-CPO, EA 7273, Université de Picardie Jules Verne, AmiensFrance

**Keywords:** lucid dream, awareness, control, prevalence, frequency, questionnaire

## Abstract

Lucid dreaming (LD) is a state of consciousness in which the dreamer is aware that he or she is dreaming and can possibly control the content of his or her dream. To investigate the LD prevalence among different samples, researchers have used different types of methodologies. With regard to retrospective self-report questionnaire, two ways of proceeding seem to emerge. In one case, a definition of LD is given to participants (“*During LD, one is–while dreaming–aware of the fact that one is dreaming. It is possible to deliberately wake up, to control the dream action, or to observe passively the course of the dream with this awareness”*), while in the other instances, participants are presented separate questions targeting specific LD indicators (dream awareness and dream control). In the present study, we measured LD frequency in a sample of French student in order to investigate for possible disparities in LD frequency depending on the type of questionnaire as outlined above. Moreover, we also study links between the prevalence of LD as assessed, respectively, by each questionnaire with various factors such as *Vividness of Mental Imagery* and *Parasomnia*. Results revealed no significant difference between LD frequencies across questionnaires. For the questionnaire with definition (DefQuest), 81.05% of participants reported experience of LD once or more. Concerning the questionnaire based on LD indicators (AwarContQuest), 73.38% of participants reported having experienced LD once or more. However, with regard to the correlations analysis, links between LD prevalence and factors such as *Vividness of Mental Imagery* and *Parasomnia*, varied across questionnaires. This result is an argument suggesting that researchers should be careful when investigating links between LD and other factors. The type of methodology may influence findings on LD research. Further studies are needed to investigate on the methodology effect in LD research namely on the respective weight of awareness and control.

## Introduction

Lucid dreaming (LD) is a state of consciousness in dreams during which the dreamer is aware that he or she is dreaming. The awareness of the dream state is a *sine qua none* condition in labeling LD (Gillespie, 1983) but this feature may be insufficient in fully assessing this phenomenon (Tholey, 1988). The possibility of controlling the dream content is cited as another core criterion of LD (Van Eeden, 1913; Snyder and Gackenbach, 1988). It is thus unclear in the literature how LD is defined (Erlacher et al., 2008; Hobson, 2009; Voss et al., 2009; Noreika et al., 2010). Moreover, there is to date no consensual method on how LD should be investigated. Snyder and Gackenbach (1988) suggested that imprecise definition can affect LD prevalence and that a multi-component definition that encompasses various elements (such as lucidity and/or the possibility of control), is needed to ensure that LD is well understood by participants. For Green and McCreery (1994) dream awareness is sufficient to define LD. Indeed, the rationale for proposing a definition which aggregates several elements of LD is questionable considering that control does not systematically occur along with dream awareness (Mota-Rolim et al., 2013; Voss et al., 2013). It is thus of upmost importance to have a consensual definition of LD since this element is crucial in devising methodologies to investigate LD.

Lucid dreaming incidence gathered in Latin America, USA, Europe, and Asia suggests that LD is a widespread phenomenon (Mota-Rolim et al., 2013) but its prevalence appears to vary across studies (cf. **Table 1**). The origin of these variations has recently been addressed by Saunders et al. (2016) in a quality meta-analysis but the authors failed to identify any explanatory systematic bias. Thus, considering that, to our knowledge, no study has directly specified the origin of variations in LD prevalence, the purpose of the present study is to address this issue by targeting the type of methodology used which is a common source of variability across studies (Schredl and Erlacher, 2004; Alvarado and Zingrone, 2008; Voss et al., 2012; Fingerlin, 2013). More precisely, we investigated experimentally whether LD prevalence would be influenced by the type of interrogation formulation (Dream Awareness and possibility of control within the same definition versus a separate evaluation for awareness and effective control).

**Table 1:** Prevalence differences of lucid dreaming (LD) across studies.

Author	Sample size	Age	Gender repartition	Country and sample	Methodology	Prevalence LD
				type		(least at once)
Schredl et al., 2016	1375	26.5 ± 18.0 years	67.42% Women	United Kingdom	Question awareness	56.32%
Schredl et al., 2012	3579	12.0 ± 1.9 years	61.36% Girls	United Kingdom	Question awareness	43.5%
Alvarado and Zingrone, 2008	492	–	68% Women	Spanish New age magazine lecturers	Question awareness	89%
Schredl and Erlacher, 2004	444	23.5 ± 5.7 years	84% Women	Unselected student sample	Definition based on awareness and control	82%
Erlacher et al., 2008	153	19.1 ± 1.1 years	60.1% Women	Japan students	Definition based on awareness and control	47%
Schredl and Erlacher, 2011	919	48.1 ± 18.4 years	54% Women	Germany representative sample	Definition based on awareness and control	51%
Erlacher et al., 2012	840	21.59 years ± 6.33	57.5% Men	German athletes	Definition based on awareness and control	56.6%
Stumbrys et al., 2013	684	25.5 ± 9.7 years	59.35% Women	German voluntaries	Definition based on awareness and control	83.5%
Smith and Blagrove, 2015	84	33.80 ± 15 years	50% Women	LD forum lecturer	Definition based on awareness and control	72.6%
Fingerlin, 2013	214	17.2 ± 1.2 years	70.6% Women	Swiss Junior college student	Definition based on awareness and control + Question LD and questions control	50%
Mota-Rolim et al., 2013	3,427	Median = 25 years	50% Women	Brazil voluntaries	Definition based on awareness and control + Question Awareness and questions control	77.2%
Voss et al., 2012	793	year range [6–19]	50% Women	German student	One-on-one Interview Description based on awareness	51.9%

**Literature search**: The purpose of this table is to illustrate how LD prevalence and methodology vary across studies focusing on LD prevalence evaluation. Titles and abstracts were searched in the electronic PubMED and PsycINFO databases and in google scholar search engine (limited to the 10 first pages) using the following search terms: lucid dream*/AND (frequency OR prevalence OR incidence). Only studies published after 2000 were examined.

Indeed, different methodologies have been devised to measure the frequency or prevalence of LD. For instance, some studies used questionnaires with a definition of LD (for example: “During LD, one is – while dreaming – aware of the fact that one is dreaming. It is possible to deliberately wake up or to control the dream action or to observe passively the course of the dream with this awareness”). This definition is then usually followed by a question on LD frequency (Schredl and Erlacher, 2004; Erlacher et al., 2008, 2012; Rak et al., 2015). In other studies, questionnaires do not contain a definition of LD and instead, propose a specific question on dream awareness. These questionnaires sometimes also cover questions on other LD dimensions, more specifically on the control of the dream content (for example: “Do you sometimes realize in your dreams that you are dreaming?” and “I am able to control or direct the content of my dreams”; Stepansky et al., 1998; Watson, 2001; Fassler et al., 2006; Soffer-Dudek et al., 2011). The effect of using these different methodologies in assessing the prevalence of LD is considered in the present study.

Using different methodologies, many researchers have tried to understand the various factors linked to LD (Blagrove and Hartnell, 2000; Patrick and Durndell, 2004; Schredl and Erlacher, 2004; Doll et al., 2009; Zink and Pietrowsky, 2013). For instance, studies have shown strong correlations between LD and dream recall frequency (Schredl and Erlacher, 2011; Jones and Stumbrys, 2014). Links between sleep characteristics and LD have been investigated with various types of retrospective questionnaires (Mota-Rolim et al., 2013). For example, it was found that lucid dreamers tend to report experiencing more spontaneous out-of-body experience than those who have not reported LD (Spanos et al., 1995; Levitan et al., 1999). Other studies have investigated the nature of the relations between LD and cognitive performances. For example, Blagrove et al. (2010) sought links between LD and Stroop task performance. In their study, lucid dreamers were able to complete the incongruent Stroop condition faster than occasional or non-lucid dreamers. Relationships have also been shown between LD and personality factors. For instance, it appears that Lucid dreamers are likely to have a more creative personality than non-lucid dreamers (Zink and Pietrowsky, 2013). Various sleep disorders have been investigated within the scope of *parasomnia*. For instance narcolepsy patients are found to report markedly higher LD frequency than typical dreamers (Dodet et al., 2015; Rak et al., 2015). Schredl and Erlacher (2004) also found an association between nightmare frequency and LD frequency. Several studies have likewise revealed links between LD and mental imagery for visual, auditory, gustatory, kinesthetic olfactory, and tactile modalities (Hearne, 1983; Kueny, 1985; Saunders et al., 2016).

For the purpose of investigating whether typical links between LD and other factors would vary depending on the type of methodology, we perform correlations between LD frequency with factors often associated with LD. We choose to focus on the following two factors: *Vividness of Mental Imagery* and *Parasomnia*.

The present study is the first to our knowledge conducted on LD prevalence using a French sample. We aimed at investigating possible disparities in LD frequency depending on the type of question form used. More precisely, the prevalence of LD was investigated with two types of questionnaires widely used in the literature: the first questionnaire contained a definition of LD and a frequency question as used by Schredl and Erlacher (2004). The second questionnaire contained two separate questions on two specific LD dimensions, one targeted the frequency of dream awareness and the other one concerned dream control. A series of questions were common to both questionnaires in order to investigate the correlation of certain factors (the *Vividness of Mental Imagery* and *Parasomnia*) with the LD prevalence. We hypothesize that the type of methodology used could have an effect on LD frequency and its correlation with *Vividness of Mental Imagery* and *Parasomnia.*

## Materials and Methods

### Participants

Participants were all students of Picardie Jules Verne University recruited from January to March 2015 through a social media website and posters pasted on the university notice boards. The term “LD” was deliberately not mentioned during the recruitment process, to ensure that participants remained blind to the purpose of the study. Participants completed a “sleep questionnaire” which lasted for approximately 35 min. Twenty participants were involved in a pre-test and were not included in the sample of the experiment. Overall, 315 participants were enrolled in the present study, 80% female and 20% male (median age: 20.8 years). An identification number corresponding to each participant guaranteed the confidentiality and anonymity of investigations. Participants were randomly assigned to two different groups.

### Material

Using the online software “google forms,” we created two questionnaires, each composed of 150 questions. The first questionnaire contained an adapted French version of a definition of LD (“*During LD, one is–while dreaming–aware of the fact that one is dreaming. It is possible to deliberately wake up or to control the dream action or to observe passively the course of the dream with this awareness*”) and a frequency question, as used by Schredl and Erlacher (2004). The second questionnaire contained two separate questions, one on the frequency of dream awareness and the other one on dream control: “*While dreaming, have you ever been aware that you were actually dreaming?*”; “*While dreaming, have you ever been able to control the content of your dream?*” These two questions were devised from existing formulations in English. They were reformulated in order to ensure a good comprehension in French language but were conceptually similar to those typically used in the literature (Stepansky et al., 1998; Watson, 2001; Fassler et al., 2006; Soffer-Dudek et al., 2011). The remaining questions were the same in both questionnaires and could be classified in the following four categories (see Annexes).

(i) *Demographics and characteristics of the participant* including 10 questions.
(ii) *Sleep quality* and *Parasomnia* (90 questions) including 24 questions from the Pittsburgh Sleep Quality Index (PSQI; Buysse et al., 1989), 61 questions from the Diagnostic Sleep Questionnaire of Hotel Dieu Paris Sleep Center (Léger et al., 2006), and 15 questions specifically devised for the present study (e.g., number of hours of sleep, frequency of waking up during the night, how rested the participant felt).
(iii) *Vividness of Mental Imagery* using 35 questions from the Psi-Q (Plymouth sensory imagery Questionnaire; Andrade et al., 2014) with five questions for each of the seven sensory modalities.
(iv) *Consumption questionnaire* including 10 questions focusing on alcohol, marijuana, caffeine, tea, soda, and cigarette consumption.

### Procedure

By clicking on the hotlink associated with the recruitment text, participants were randomly redirected to one of the two questionnaires. A PHP/HTML page hosted on a personal web server managed the random distribution. After completion of the questionnaire all the answers marked with a timestamp were created in an online spreadsheet. The data were then transferred to an Excel spreadsheet where we excluded duplicated data and incomplete responses.

### Data Acquisition and Pre-processing

As a reminder, the purpose of the present study is (1) To investigate whether the prevalence of LD would vary depending on the type of methodology and (2) To study links between the prevalence of LD as assessed, respectively, by each questionnaire, with the following two factors: Vividness of Mental Imagery and Parasomnia.

Analysis would thus concern only items of the Mental Imagery scale and the 10 questions on parasomnias that could be remembered by participants at wake. Exploratory items were not considered in the present statistical analysis (for example, questions on sleep position, consumptions, duration of sleep…).

For the questionnaire in which a definition of LD was presented (DefQuest), LD frequency per week was measured with an 8-point rating scale ranging from zero (never) to seven (several times). For the other questionnaire which contained a question about the frequency of dream awareness and about dream control (AwarContQuest), awareness and dream control were both, respectively, measured with a 6-point rating scale ranging from zero (never) to five (several times), to assess the frequency of each manifestation per week. To obtain unit in frequency per month, the scales were recoded using the Schredl and Erlacher (2004) methodology.

For *parasomnia* category (PSQI; Buysse et al., 1989), we selected 10 questions about the following: “headaches”; “kicks”; “hypnagogic hallucinations”; “immediate dreams”; “paralysis”; “nightmares”; “coughs”; “gastric burns”; “ruminations”; “narcolepsy.” These 10 variables were evaluated on a 6-point frequency scale ranging from zero (never) to six (every day). A total parasomnia score was calculated by summing up the point for each 10 responses.

For *Vividness of Mental Imagery*, we calculated seven scores corresponding to the seven imagery modality subscales “vision,” “audition,” “smell,” “taste,” “touch,” “body,” and “emotion” in 11-point intensity scale (Andrade et al., 2014). A *Vividness of Mental Imagery* score was calculated by summing up the point for each seven subscales score.

## Results

Data collection and processing was carried out using SPSS^®^ version 20 for Windows. Non-parametric tests were conducted since the conditions of homogeneity and normality of variances were not met. After exclusion of contributions with missing answers, the statistical analyses concerned 309 participants out of the original sample of 315 individuals.

**FIGURE 1:**
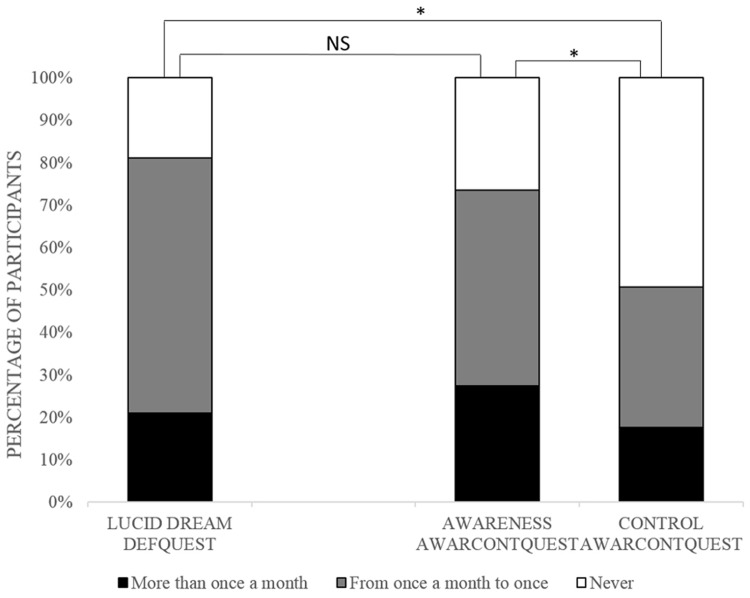
**Percentage of participants reporting LD in DefQuest (*N* = 154) and awareness and control in AwareContQuest (*N* = 153)**.

**FIGURE 2:**
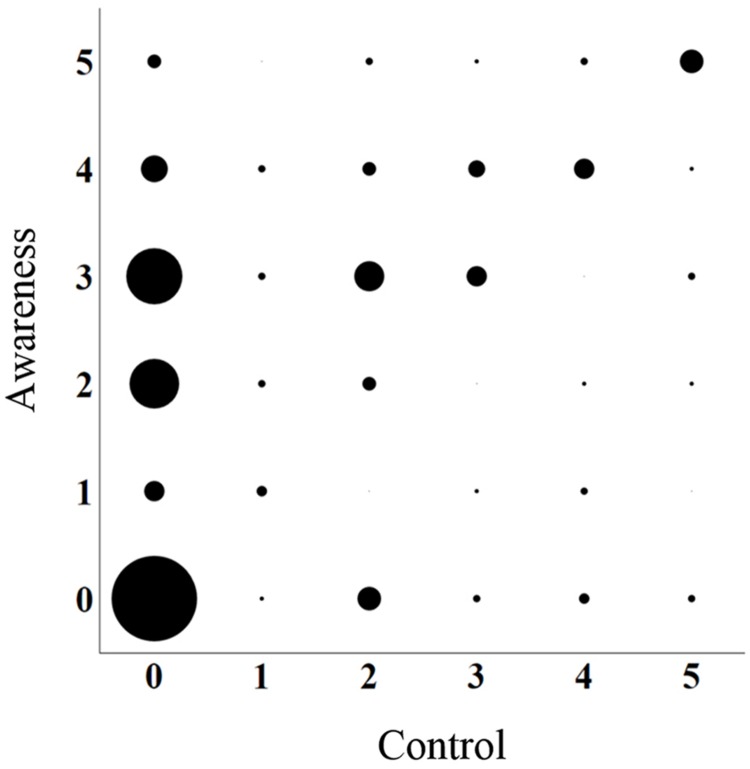
**The size of the black circles represents the number of participant responding to both questions (awareness and control) in AwareContQuest.** Awareness and control were both evaluated on the same 6-point rating scale (0: never, 1: once, 2: less than once a year but more than once, 3: many times a year, 4: many times a month, 5: many times a week).

As shown in **Figure 1**, among participants who have answered the DefQuest (*n* = 153), 81.05% reported having experienced LD at least once. Among participants who answered the AwarContQuest (*n* = 154), 73.38% reported having experienced dream awareness at least once. Concerning the dream control question, 50.65% reported dream control at least once. For the AwarContQuest, among the 113 participants who reported one dream or more with awareness, 79 reported a lower frequency for dream control. Moreover, among the 76 participants who reported one experience of dream control or more, 23 also reported a low frequency of dream awareness (**Figure 2**).

No significant difference was found between LD frequency (DefQuest) and dream awareness frequency (AwarContQuest) on the Mann–Whitney test. However, a significant difference (*p* < 0.001) appeared between LD frequency (DefQuest) and dream control frequency (AwarContQuest). The Wilcoxon signed rank test was significant (*p* < 0.001) between dream awareness and dream control in the AwarContQuest.

Using Mann-Whitney test, we also performed a comparison of scores between the two questionnaires (DefQuest vs. AwarContQuest) and the *Vividness of Mental Imagery* score. For the *Parasomnia* score, we used a chi-Square to perform comparisons. No significant difference was found for all these comparisons, except for *Vividness of Mental Imagery* in vision modality (**Table 2**).

**Table 2:** Descriptive data and significance of the Chi-square and Mann-Whitney tests between the two experimental groups (DefQuest and AwareContQuest).

Type of questionnaire	DefQuest *N* = 153	AwareContQuest *N* = 154	Sig.
**Demographic**			
Gender women/men	123/30	124/30	NS
Age mean and (*SD*)	20.27 (2.38)	20.11 (1.98)	NS

***Vividness of mental imagery* score**			
Mean and (*SD*)			
“Vision”	7.58 (1.54)	7.34 (1.43)	0.04
“Sound”	7.37 (1.85)	7.04 (1.94)	NS
“Smell”	5.73 (2.4)	5.68 (2.23)	NS
“Taste”	6.15 (2.38)	6.06 (2.47)	NS
“Touch”	6.91 (2.35)	6.94 (2.33)	NS
“Body”	6.74 (2.11)	6.75 (1.99)	NS
“Emotion”	6.96 (1.73)	6.75 (1.9)	NS
Score	47.44 (12.16)	46.56 (12.14)	NS
**Parasomnia score**			
Mean and (*SD*)			
“Headache”	1.81 (1.57)	1.69 (1.52)	NS
“Kicks”	2.14 (1.88)	2.08 (1.85)	NS
“Hyp. hallucinations”	1.01 (1.60)	1.06 (1.58)	NS
“Immediate dreams”	2.27 (1.95)	2.07 (1.92)	NS
“Sleep paralysis”	0.77 (1.51)	0.82 (1.42)	NS
“Nightmare”	2.29 (1.39)	2.16 (1.34)	NS
“Cough”	1.48 (1.30)	1.79 (1.33)	NS
“Gastric burn”	0.61 (1.14)	0.49 (1.09)	NS
“Rumination”	3.42 (1.44)	3.44 (1.48)	NS
“Narcolepsy”	2.06 (1.82)	1.97 (1.68)	NS
Score	17.07 (6.94)	16.56 (7.23)	NS

### Correlations

We conducted a Holm-Bonferroni sequential correction (Holm, 1979; Gaetano, 2013). Spearman’s rho correlation was used to explore the relationship between LD frequency (in DefQuest), awareness and dream control frequencies (in AwarContQuest) and the two factors (*Parasomnia Vividness of Mental Imagery)*. *Parasomnia* score correlated with dream awareness, *r*(152) = 0.200, *p* = 0.028, and dream control, *r*(152) = 0.263, *p* = 0.002, in the AwarContQuest, but not with LD frequency in DefQuest. *Vividness of Mental Imagery* score correlated with dream control *r*(152) = 0.189, *p* = 0.019, in AwarContQuest, but neither with dream awareness frequency in AwarContQuest nor with LD frequency in DefQuest.

## Discussion

The present study aims at investigating for possible disparities in LD frequency depending on the type of methodology. We thus investigated the prevalence of LD with two questionnaires: the *DefQuest* contained a definition of LD and a frequency question and the *AwarContQuest* contained two separate questions on two dimensions of LD, one about the frequency of dream awareness and the other about dream control. A series of questions were, however, common to both questionnaires to investigate whether the correlation of certain factors (*Parasomnia* and Vividness of Mental Imagery) with the LD prevalence, could vary depending on the type of questionnaire used.

### Prevalence of LD as a Function of the Type of Questionnaire

The prevalence of LD was 81.05% when the definition of LD was given. On the other hand, without a definition, prevalence of dream awareness and dream control, were, respectively, 73.38 and 50.65%. Contrary to our expectations, no major discrepancies on LD frequency were observed using different methodologies in our study. Indeed, the prevalence of LD obtained with *DefQuest* was not statistically different to the awareness frequency in *AwarContentQuest*. Control frequency was different to awareness frequency in the AwarContQuest. The finding that dream control is not exclusive or systematic to dream awareness, is not a new finding (Voss et al., 2013). It thus seems that these two components of the LD definition, awareness and control, may be at least in part independent features. It is therefore problematic in methodologies such as the *DefQuest*, to identify what the participant has considered in the definition of LD (awareness OR/AND control?).

Authors have advised the use of an example to illustrate LD and to bring clarity to the given definition (Snyder and Gackenbach, 1988; Schredl and Erlacher, 2004). LD is a complex phenomenon, which as in the present study, does not systematically occur along with both awareness and control (Voss et al., 2013). Several proposals have been made for more adequate methodologies to investigate LD. For example, hybrid strategies have been employed, bearing in this way the respective benefits of both types of questionnaires used in our study (DefQuest and AwarConQuest; Snyder and Gackenbach, 1988; Voss et al., 2013). Fingerlin (2013) conducted research in which a definition of LD was presented along with the frequency scale used in Schredl and Erlacher (2004). However, for a more precise measurement of LD prevalence, Fingerlin (2013) also added the following question “*I had one or several dreams meeting only one of the first two criteria.*” In a more recent study, Mota-Rolim et al. (2013) proposed a definition of LD but in addition, they added distinct questions on LD frequency and control frequency. In another study, Mota et al. (2016) used an interesting methodology where LD awareness and dream control are considered separately: “*Can you be aware of dreaming during sleep*?” “*Can you control your dream when this happens*?”

Among the various methods used to investigate dream characteristics, dream mentation report can be an interesting paradigm that could be adapted to LD research (Stickgold et al., 1994; Windt, 2013; Speth et al., 2015; Speth and Speth, 2016a). Dream mentation report may differ from typical (narrative) dream report by considering broader subjective mentation occurring prior to waking. Mentation report can be elicited by specific questions for instance: *“****When you awaken, think back and try to remember what was going on in your mind in the time prior to waking.”*** (Speth and Speth, 2016b). Typically, the responses of participants are then analyzed by the experimenter (McNamara et al., 2005).

Adjusting such methodology to the specific case of LD research, would reduce the strong reliance on participants in identifying LD. Indeed, the task of stating whether a dream is lucid or not, would be performed by the experimenter based on his or her definition of LD and not on what the participant would consider as a LD. Moreover, using a double or multiple rating procedure, could further improve the confidence in the identification of LD. The use of dream mentation report could thus be a promising methodology but the generalization of results with such technic, would be possible only if a consensual definition of LD is used in the literature.

However, methodologies such as dream report (or dream mentation report) analysis also trigger new interrogations. By requesting participants to response to specific questions in order to collect information on their dreams, we cannot exclude that their recollections could be affected by these cueing questions.

Various methods are available for investigating LD and other alternatives can also be devised for evaluating this phenomenon. However, it is important to be aware of the forces and weaknesses of each methodology and most importantly, we should also be able to state clearly what a given method measures specifically. Saunders et al. (2016), in a quality meta-analysis, have released a tool for measuring the methodological quality of studies in LD prevalence: the “*LD Incidence Methodological Quality Scale*” (LDIM-Qi). The LDIM-Qi advocates the need of a clear definition that does not focus on control as a necessity. It also advices the adjunction of a LD example, the asking of a narrative recall of LD from the participants, a clear question wording, the control of confounding factor (such as social desirability) and a 7+ point clear scale.

### Correlations between LD and Other Factors

We also investigated whether the correlation of *Vividness of Mental Imagery* and *Parasomnia* with the prevalence of LD could vary depending on the type of questionnaire used. LD frequency in the DefQuest did not correlate with neither *Vividness of mental imagery* nor *Parasomnia* score. In the AwarContQuest, both *Parasomnia* score and *Vividness of Mental Imagery* score correlated with control and *Parasomnia* score correlated with awareness.

Differences between LD frequency obtained with DefQuest and AwarContQuest are not apparent but, all things being equal, the interesting information here is that the two types of methodology induced different correlations. If these results can be replicated, future research will have to control systematically if the participant considered the awareness or the control of LD when presenting a multifactorial definition. Historically, the motivation for proposing a definition of LD that encompasses various factors, is to ensure a clear understanding of LD and to avoid confusion with “*morning-after dream recall*” (Snyder and Gackenbach, 1988). However, in the light of the present study, presenting a broad definition to investigate a unique frequency indicator, may induce ambiguity regarding the respective prevalence of awareness and control in LD.

## Conclusion

In the present study, we measured LD frequency in a sample of French student in order to investigate for possible disparities in LD frequency depending on the type of methodology. We also study links between the prevalence of LD as assessed, respectively, by each methodology with factors such as *Vividness of Mental Imagery* and *Parasomnia*. Results revealed no significant difference between LD frequencies across methodologies. However, with regard to the correlations analysis, links between LD prevalence and factors such as *Vividness of Mental Imagery* and *Parasomnia*, varied across questionnaires. If these findings are confirmed, our study tends to suggest that the type of methodology may affect correlations between LD and other factors (such a mental imagery). Regarding the assessment of LD prevalence, it appears that the type of methodology cannot explain the variability of LD frequency across studies. Others factors such as age (Schredl et al., 2012; Voss et al., 2012), cultural representations toward dream experience (Erlacher et al., 2008; Mota-Rolim et al., 2013) or the fact that retrospective measurement is dependent on memory and meta-cognitive capacity (Mota-Rolim et al., 2013; Aspy et al., 2015), have already been pointed out to possibly explain this discrepancy. However, further studies are still needed to investigate the respective contribution of each of these factors in generating variability in LD frequency. Prior to these investigations, the proposal of a more accurate and consensual definition of LD with the appropriate methodologies, is needed.

## Author Contributions

VQ supervised the whole research. NR constructed the research protocol and collected all the data. YG participated in the writing of the manuscript and in interpreting the results.

## Conflict of Interest Statement

The authors declare that the research was conducted in the absence of any commercial or financial relationships that could be construed as a potential conflict of interest.
